# Prenatal Phthalates Exposure and Cord Thyroid Hormones: A Birth Cohort Study in Southern Taiwan

**DOI:** 10.3390/ijerph18084323

**Published:** 2021-04-19

**Authors:** Po-Chin Huang, Pao-Lin Kuo, Wei-Hsiang Chang, Shu-Fang Shih, Wan-Ting Chang, Ching-Chang Lee

**Affiliations:** 1 National Institute of Environmental Health Sciences, National Health Research Institutes, Miaoli 350, Taiwan; pchuang@nhri.edu.tw (P.-C.H.); wtchang2@nhri.edu.tw (W.-T.C.); 2 Research Center for Environmental Medicine, Kaohsiung Medical University, Kaohsiung 807, Taiwan; 3 Department of Medical Research, China Medical University Hospital, China Medical University, Taichung 406040, Taiwan; 4 Department of Obstetrics and Gynecology, Medical College, National Cheng Kung University, Tainan 701, Taiwan; paolinkuo@gmail.com; 5 Department of Food Safety/Hygiene and Risk Management, National Cheng Kung University, Tainan 701, Taiwan; whchang@mail.ncku.edu.tw; 6 Research Center of Environmental Trace Toxic Substances, College of Medicine, National Cheng Kung University, Tainan 701, Taiwan; 7 Department of Health Administration, College of Health Professions, Virginia Commonwealth University, Richmond, VA 23284, USA; shihs2@vcu.edu; 8 Department of Environmental and Occupational Health, Medical College, National Cheng Kung University, Tainan 701, Taiwan

**Keywords:** phthalate metabolites, thyroid hormone, cord blood, birth cohort

## Abstract

Background: The regulation of thyroid hormones in the early stages of gestation plays a crucial role in the outcome of a pregnancy. Furthermore, thyroid hormones are fundamental for the fetal development of all organs, including endocrine hormone changes in uterus. Endocrine disrupting chemicals have been shown to have an effect on thyroid hormone homeostasis in newborns, which affects their later development. Few studies have proposed how phthalates could alter thyroid function through several mechanisms and the possible effects on thyroid hormone homeostasis of phthalates on pregnant women. However, the effects of cord blood phthalates and prenatal phthalate exposure on thyroid hormones in newborns remain unclear. Objectives: We aim to follow up on our previous established subjects and determine the correlation between phthalate exposure and thyroid hormones in pregnant women and newborns. Materials and methods: We recruited 61 pregnant women from the Obstetrics and Gynecology Department of a medical hospital in southern Taiwan and followed up. High performance liquid chromatography electrospray ionization tandem mass spectrometry (HPLC-ESI-MS/MS) was used to analyze urine samples for five phthalate metabolites. Serum levels of thyroid hormones were analyzed using electrochemoluminescence immunoassay (ECLIA) method. We used Spearman and Pearson correlation coefficients to evaluate the correlation between each phthalate metabolites in serum and the thyroid hormone levels in fetus and parturient. Finally, multiple logistic regression was used to explore the relationship between hormones and their corresponding phthalate metabolites in cord blood. Results: High MBP in cord blood was correlated with negative cord serum TSH in newborns (r = −0.25, *p* < 0.06). By using multiple linear regression after adjusting for potential confounders (gestational and maternal age), cord serum MBP levels showed a negative association with cord serum TSH (β = 0.217, *p* < 0.05), cord serum T_4_ (β = 1.71, *p* < 0.05) and cord serum T_4_ × TSH (β = 42.8, *p* < 0.05), respectively. Conclusion: We found that levels of cord serum TSH and T_4_ in newborns was significantly negatively associated with cord serum MBP levels after adjusting for significant covariate. The fall in TSH in newborns may potentially be delaying their development.

## 1. Introduction

Thyroid hormone is fundamental for fetal development of all organs, including endocrine hormone changes in uterus. Some endocrine disruptor chemicals (EDCs) have been reported to have a possible effect on thyroid hormone homeostasis in newborns, such as polychlorinated biphenyl (PCB), or polybrominated diphenyl ethers [1,2,3], persistent organic pollutants (POP) [4] and bisphenols [5]. Permanent effects of thyroxin-related development, like on neurons in the brain, in infants and in later childhood are observed clinically where hypothyroidism of pregnant women occurred during pregnancy [6,7,8,9,10,11,12]. Previous studies had revealed possible effects on thyroid hormone homeostasis while exposed to certain phthalates in pregnant women [13,14,15]. A few studies reported the adverse or positive relationship between levels of cord serum phthalate metabolite and cord serum thyroid hormone (e.g., TSH and thyroxine) [15,16,17], however, whether phthalate exposure in the uterus can cause thyroid hormone alterations in newborns was debatable due to limitations such as small sample size, spot urine or serum sample, different phthalates or thyroid hormones observed, etc.

Phthalates are ubiquitous in daily life. They are added to plastics and many other daily products [18,19]. From 2003 to 2007, an average of 200,000 tons of di (2-ethylhexyl)phthalate (DEHP) and 20,000 tons of dibutyl phthalate (DBP) were used to produce consumer products in Taiwan [20]. Although phthalates are metabolized to their metabolites within a few hours or days [21], the potential consequences of human exposure to phthalates have focused on susceptible subjects, like pregnant women and fetuses [22,23,24,25,26,27,28]. Phthalate metabolites are considered to be good biomarkers for evaluating phthalate exposure in humans because of their low contamination rate in the laboratory and reliability for indicating an individual’s phthalate exposure [18,21,29]. In addition, animal and epidemiological studies have reported that phthalate metabolites can penetrate the placenta and be retained in the fetus [14,30,31,32]. Biomarkers of phthalates in different specimens were used to assess the exposure of early life in the uterus, such as meocoin, serum, and amniotic fluid samples [14,21,33].

Phthalates have also been suggested as having a possible antagonistic effect on thyroid functions [34,35,36,37,38,39,40] and may alter thyroid hormone through the oxidative stress pathway [19,41,42]. This may be relevant to other environmental disrupter compounds [1,40,43,44]. Though several epidemiological studies have investigated the association between phthalate metabolites and maternal and cord serum thyroid hormones, there were inconsistencies in the observed results of specific phthalates and the alterations of the phthalate-thyroid hormone relationships [16,17,45,46,47]. Furthermore, little is known about the maternal phthalate metabolites in urine, serum and cord blood samples at delivery, in relation to maternal serum and cord serum thyroid hormones.

Therefore, the aims of this study are to investigate the relationship between phthalate metabolites and thyroid hormones in cord serum and maternal serum samples using the existing cohort we established.

## 2. Material and Method

### 2.1. Participants

Participants of this study were recruited from a cohort for evaluating prenatal phthalates exposure (during the third trimester) to the pregnant women and newborns during 2005 to 2006 [13]. All participants were interviewed and the benefits and risks of participating in this longitudinal project were fully explained. Of all participants who signed the informed consent for this study, 76 pregnant women received follow-up, and urine, serum and cord blood samples were collected in 61 of them. All samples were collected in the third trimester before delivery. The protocol was approved by the Human Ethics Committee of the National Cheng Kung University Hospital.

### 2.2. Samples Collection

Urine samples of 20–30 mL were collected using 250 mL glass vessels and the urine samples were immediately transferred into 12 mL amber glass bottles for phthalate monoester and creatinine analysis. All urine samples were stored at −20 °C until analysis. Meanwhile, we drew 8 mL blood samples via venipuncture into chemically clean glass tubes containing no anti-coagulant. After delivery, cord blood samples were drawn by gynecologists using 20-mL glass syringe and transferred into chemically clean glass tubes. Maternal and cord blood were centrifuged at 2500 rpm in 45 min to obtain serum samples and stored at −70 °C in amber glass bottles until analysis. To prevent possible contamination of the urine and serum samples, all the glassware had been washed in methanol, acetonitrile and acetone, and then was sealed with aluminum foil before sample collection. Glass syringes were sterilized with ethylene oxide for the cord blood sample collection. We used 5 mL of HPLC-grade H_2_O to extract all the glassware, and it was analyzed to ensure no phthalate metabolites contamination during the preparation of the glassware.

### 2.3. Phthalate Metabolites Analysis

We used a previously described analytical method to determine phthalate monoester levels in urine samples [13]. We made some modifications to the previous method in the analytical column for serum phthalate metabolites analysis [48]. Briefly, we used high performance liquid chromatography electrospray ionization tandem mass spectrometry (HPLC-ESI-MS/MS), to analyze the level of urine samples for five phthalate metabolites: monobutyl phthalat (MBP), mono-benzyl phthalate (MBzP), mono-2-ethylhexyl phthalate (MEHP), mono-ethyl phthalate (MEP) and monomethyl phthalate (MMP). The limits of detection (LOD) of five phthalate metabolites were 1.4 ng/mL (MBP), 1.4 ng/mL (MBzP), 0.9 ng/mL (MEHP), 1.0 ng/mL (MEP) and 1.4 ng/mL (MMP).

### 2.4. Assay for Maternal Serum and Cord Serum Thyroid Hormones

Maternal serum and cord serum thyroid hormones, which include triiodothyronine (T_3_), thyroxin (T_4_), free T_4_ (FT_4_), and thyroid stimulating hormone (TSH), were analyzed using combined clinical chemistry and immunoassay tests (Modular Analytics Serum Work Area; Roche Diagnostics) and an electrochemoluminescence immunoassay (ECLIA) (Elecsys 2010 and Modular Analytics E170; Roche Diagnostics), respectively. Urinary creatinine level was re-analyzed and re-confirmed if the level exceeded the reference range.

### 2.5. Physical Examination of Health Status in Newborns

Physical examination and measurements of the newborns were done and recorded by the same pediatrician and a well-trained assistant. The measurements included the newborns’ birth anthropometric measurements, AGD and gestational age. To obtain an average AGD for each infant, AGD were measured twice. For female newborns, the AGD was measured from the center of the anus to the posterior convergence of the fourchette and to the junction of perineal skin with the rugated skin of the scrotum for male newborns [49].

### 2.6. Statistical Analysis

All statistical analysis was performed using SPSS 22.0 (IBM, Armonk, NY, USA). All the measured phthalate metabolites in maternal urine, serum and cord blood were log-transformed to approximate normal distribution. Covariate selection (e.g., age and gestational age, cigarette smoking, sex, birth weight, etc.) was based on the results of relevant studies [15,50]. We used Spearman and Pearson correlation coefficients to evaluate the correlation between each phthalate monoester in serum and thyroid hormone levels in fetus and parturient. We also used multiple linear regression to assess the associations among cord serum phthalate metabolites and cord serum thyroid hormone levels in newborns, adjusting for potential confounders in the forward stepwise regression model.

## 3. Results

### 3.1. Demographic Characteristics of Participants and Physical Examination of Newborns

The mean age of the participants was 34.0 ± 3.5 years (range: 26–43 years). The average gestation age at delivery was 39.0 ± 1.2 weeks. All our participants were non-smokers, but 11 participants had been exposed to passive smoke (18.3%). None of them were an “alcohol drinkers”, which was defined as “someone who consumed any alcohol at all during pregnancy”. No significant differences was observed between the levels of urinary phthalate metabolites and smoking habits and drinking. From the 76 initially recruited pregnant women, 61 foetuses were followed until birth. Significant differences between birth length, AGD and AGI-W were observed between male and female newborns; birth length (*p* < 0.01) and AGD (*p* < 0.01) were longer in males than females (Table 1).

### 3.2. Phthalate Metabolites in Maternal Urine, Serum, and Cord Blood

The detectable rates of MBP, MEHP, MEP, MMP and MBzP in all urine samples were 100%, 100%, 98%, 52% and 19%, respectively. Median levels without creatinine adjustments for five urinary phthalate metabolites at delivery were 114 ng/mL (25.4–1830) for MBP, 40.2 ng/mL (3.6–958) for MEHP, 36.4 ng/mL (ND-1980) for MEP, 8.3 ng/mL (ND-169) for MMP, and 5.7 ng/mL (ND-218) for MBzP (Table 2). Amongst the five urinary phthalate metabolites levels, MBP, MEP and MEHP were the highest, which suggests the predominant exposure to phthalates DBP, DEHP and DEP of our participants. The proportions of MBP, MEHP and MEP of total phthalate exposure were 59%, 18% and 16%, respectively.

The detectable rates of MBP and MEHP in all serum and cord serum samples were 100%, whereas MEP, MMP and MBzP were detected in less than 10% of all samples. Median levels of five phthalate metabolites in maternal serum at delivery were 158.0 ng/mL (59.6–1080) for MBP, 21.0 ng/mL (9.2–99.2) for MEHP, 2.8 ng/mL (ND-26.5) for MEP, ND ng/mL (ND-3.7) for MMP and ND ng/mL (ND-10.1) for MBzP (Table 2). Levels of MBP and MEHP in maternal serum were the highest of the five metabolites measured, which contributed over 95% of total phthalate exposure in pregnant women. The proportions of MBP and MEHP of total phthalate exposure in maternal serum were 87% and 11%, respectively.

In addition, the median levels of the five phthalate metabolites in cord serum were 256.0 ng/mL (65.2–815) for MBP, 24.7 ng/mL (11.0–665.0) for MEHP, ND ng/mL (ND-9.3) for MEP, ND ng/mL (ND-13.3) for MMP and ND ng/mL (ND-26.8) for MBzP (Table 2). Contribution profiles of MBP and MEHP in cord serum were quite similar to those in maternal serum. The proportions of MBP and MEHP of total phthalate exposure in cord serum were 90% and 9%, respectively.

### 3.3. Thyroid Hormone Levels in Pregnant Women and Newborns

We have compared the thyroid hormone levels of our participants to that of the general Taiwanese population, since there is no thyroid hormone reference range available for pregnant women and newborns. From our data, it is observed that more than 90% of T_3_, T_4_ and TSH levels in maternal serum samples were within the reference range of the general Taiwanese population. The low FT_4_ levels in our participants (more than 35% are lower than the lowest level of the general population) might suggest a possible mild thyroxine insufficiency (i.e., hypothyroidism). In addition, median levels of cord serum TSH and FT_4_ were higher than those in maternal serum (Table 3), whereas maternal serum T_4_ and T_3_ levels were much lower in the fetus. Although there is one outlier (hypothyroidism) which is excluded in the following analysis, the distribution of thyroid hormones in maternal serum and cord serum were not significantly changed.

### 3.4. Association between Phthalate Metabolites in Maternal Serum, Cord Serum and Thyroid Hormones

**Figure 1:**
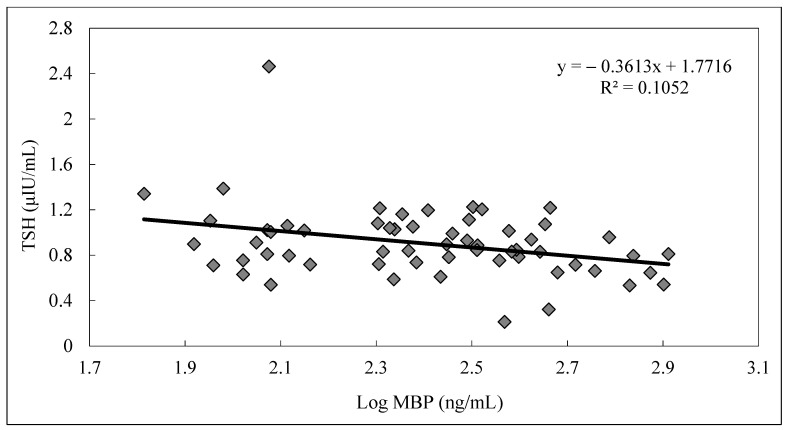
Relationship between log MBP levels and TSH levels in cord serum samples (*n* = 60).

For maternal serum samples, significantly positive correlations were observed between levels of maternal serum T_4_ and FT_4_ (R = 0.76, *p* < 0.05), maternal serum T_4_ and T_3_ (R = 0.53, *p* < 0.05) and levels of maternal serum T_3_ and TSH (R = 0.39, *p* < 0.05) for pregnant women. For cord serum sample, significant positive correlation was also observed between levels of cord serum T_4_ and FT_4_ (R = 0.62, *p* < 0.05), and cord serum T_4_ and cord serum TSH (R = 0.35, *p* < 0.05) in newborns. However, no significant correlations were found between phthalate metabolites and thyroid hormones in maternal serum samples. In addition, a marginally significant negative trend between cord serum MBP and cord serum TSH (R = 0.25, *p* = 0.058), and cord serum MBP and cord serum T_4_ (R = 0.23, *p* = 0.092) was observed (Table 4). As cord serum MBP level increased, a decreasing trend of cord serum TSH in newborns was also observed (Figure 1).

### 3.5. Regression Analysis

A multiple regression model was used to examine the association between thyroid hormone level and phthalate metabolites in cord serum (Table 5). After adjusting for gestational age and maternal age (sex, cigarette smoking, birth weight and cord serum MEHP were excluded in the stepwise forward model), cord serum MBP levels showed a negative association with cord serum TSH (TSH: β = −0.217, *p* < 0.05), cord serum T_4_ (β = −1.71, *p* < 0.05) and cord blood TSH × T_4_ (β = −42.8, *p* < 0.05); however, we found a positive correlation between cord serum MBP and cord serum FT_4_/T_4_ (β = 0.036, *p* < 0.01).

## 4. Discussion

In this study, we found a correlation between higher exposure levels of cord serum phthalate and alterations in cord serum thyroid hormones in newborns. Despite having small sample size, the association between higher cord serum MBP level and low cord serum TSH and cord serum T_4_ remained after controlling for other variables in multiple regression model.The urinary phthalate metabolites levels in this study are consistent with our previous study [13], where we found that Taiwanese women (2005–2006) are exposed to a higher level of phthalates than the average American pregnant women [25], whereas their levels dropped dramatically after the 2011 DEHP scandal [15,42].

Some toxicological studies have shown possible thyroid hormone antagonist activities of certain phthalates, such as DBP and DEHP in adult animals [34,36,37,41]. Little information is available about phthalate exposure in the uterus and its effects on fetal thyroid. A two-generation study was conducted to evaluate the synergetic effect of PCB and DEP on adrenal and thyroid glands in rats. Follicular shrinkage, loss of thyroglobulin and fibrosis of the interfollicular epithelium was found in both treated parental and F1-generation male and female rats [38]. Another animal study has observed the morphological changes of the thyroid gland through the effect of DEHP [40].

Some possible mechanisms explaining how phthalates may alter thyroid hormones have been studied in experimental studies. Assessment of T_3_-antagonist activity using a thyroid hormone assay of three phthalates including BBzP and DBP done by a previous study showed TH-antagonist activities in vivo [37]. In addition, an investigation into the effects of six phthalates on transcriptional activity of sodium/iodide symporter (NIS) showed that DBP appeared to downregulate the human NIS promoter [43]. This suggested that phthalates such as DBP and DEHP could modulate transcriptional activity to induce thyroid hyperactivity and decrease the concentration of thyroxin. Besides, DEHP can perturb thyroid hormone homeostasis and reduce thyroid hormone levels through the activated Ras/Akt/TRHr pathway in thyroid-disrupting effects of DEHP [41].

Epidemiological studies [13,14,15,45,47,50] have shown possible effects on thyroid hormone homeostasis in humans. Some studies have reported that certain phthalate metabolites, such as MBzP, were inversely associated with cord serum TSH [16,17]. Phthalates indexes were also inversely associated with cord serum TSH and total T_4_ [17]. However Yao et al. did not observe any associations between urinary phthalate concentration and cord sera thyroid hormone [47]. Since phthalates can penetrate placenta [14,30,31,32] and clear clinical evidence of low maternal thyroid can affect thyroid function in newborns [51,52,53,54], it reveals that some phthalates, like DBP, may mimic functional thyroxin and cause a mildly decreased level of TSH in newborns. However, Minatoya et al. did not find any adverse effects of thyroid hormone levels in infants with prenatal DEHP exposure [46]. The discrepancies in results observed might be due to the possible differences in time of sample collection.

In this study, cord serum phthalate metabolites were observed to be higher than those in maternal serum. This might not indicate a possible placenta penetration of phthalate metabolites such as MBP and MEHP because we did not observe correlations between each pair of phthalate metabolites in maternal serum and cord serum. Hence, more research is needed to understand the possible underlying association and mechanism in the uterus. The knowledge of placenta transportation and metabolic ability of phthalates in uterus in animal studies [32] is much clearer than in humans. A previous study showed that DEHP and MEHP can penetrate the placenta [30] and some studies [55,56,57] have reported that phthalate metabolites existed in human serum as both a free and conjugated form. In addition, phthalates have also been detected in the urine of newborns [58]. Therefore, phthalates and phthalate metabolites may penetrate placenta by different mechanisms. These mechanisms may seem unclear, however it is possible that phthalate metabolites may penetrate placenta in its free form and accumulate in the fetus in its conjugated form. However, further studies are still needed to clarify this phenomenon.

Since more evidence showed the possible effects on thyroid hormone homeostasis for certain phthalates in animal and epidemiological studies [13,14,15,34,35,36,37,38,40,41,45,51], we cannot rule out the possible effect phthalate exposure has on thyroid function and other hormones [59,60,61]. Further research is still needed to clarify the possible mechanisms of such effect.

In addition to the limitations of this study previously described would be the small sample size and limited number of phthalate metabolites being analyzed [13], we did not measure the secondary metabolites of DEHP in this study. While we took precautions to prevent contamination during collection and analysis of serum samples, levels of phthalate metabolites in cord blood were higher than in maternal serum and distinguished profiles of phthalate metabolites in urine and serum samples were found (Figure 1). Urinary MBP, MEHP and MEP contributed over 95% of total phthalate exposure, whereas MBP and MEHP were dominantly compounds in serum and cord blood samples. Short-chain phthalates, like DEP and DMP, were rapidly metabolized to their metabolites in a few hours [62], which instantly excreted to urine and may not cause significant placenta transportation [63,64]. For long-chain phthalates with longer half-lives, like DBP and DEHP, continuous exposure to these phthalates through food and food packaging materials [18,65] are possible reasons that MBP and MEHP were both dominant compounds in serum and urine samples.

## 5. Conclusions

We found that the level of cord blood TSH in newborns was significantly negatively associated with MBP levels in cord blood after adjusting for covariates. The fall in TSH levels in newborns may be potentially delaying their development. Hence, questions about the relationship between thyroid and testosterone hormones in the uterus are needed for further investigation.

## Figures and Tables

**Table 1:** Physical examination of health status in newborns (*n* = 61) ^a^.

Newborns’ Health Status	Males (*n* = 31)	Females (*n* = 30)	*p*-Value ^b^
Birth weight (g)	3250	3087	
	(1678–4260)	(2120–3935)	0.055
Birth length (cm)	50.4	48.7	
	(42.0–56.0)	(44.1–53.5)	<0.01 *
Gestational age (weeks)	39.1	38.7	
	(35.3–41.7)	(35.8–41.4)	0.072
AGD (mm) ^a^	22	17	
	(12–36)	(7–23)	<0.01 *

^a^ The anogenital distances of one female and two male newborns were not available because of conducting blood infusion in the NICU. AGD = anogenital distance. ^b^ Wilcoxon rank sum test, * *p* < 0.05.

**Table 2:** Concentrations of phthalate monoesters in urine, serum and cord blood in the third trimester before delivery (ng/mL, *n* = 61).

Phthalate Monoesters	Urine		Serum		Cord Blood	
	Median (Range)	10–90th	Median (Range)	10–90th	Median (Range)	10–90th
MBP ^a^	114 (25.4–1830)	36.9–550.6	158.0 (59.6–1080)	64.9–413.0	256.0 (65.2–815)	97.4–604.8
MEHP	40.2 (3.6–958)	8.4–152.0	21.0 (9.2–99.2)	11.7–37.1	24.7 (11.0–665.0)	14.2–65.9
MEP	36.4 (ND ^b^-1980)	4.6–236.8	2.8 (ND-26.5)	ND-6.3	ND (ND-9.3)	ND-3.4
MBzP	5.7 (ND-218.0)	1.9–49.2	ND (ND-10.1)	ND-2.8	ND (ND-26.8)	ND-3.6
MMP	8.3 (ND-169)	1.7–38.0	ND (ND-3.7)	ND-2.4	ND (ND-13.3)	ND-ND

^a^ MBP = monobutyl phthalate; MBzP = monobenzyl phthalate; MEP = monoethyl phthalate; MEHP = mono-2-ethylhexyl phthalate; MMP = monomethyl phthalate. ^b^ Detection limit (LOD) of phthalate monoesters were: MBP, 1.4; MBzP, 1.4; MEP, 1.0; MEHP, 0.9; MMP, 1.4 ng/mL, respectively. Half of LOD was calculated as the detected value below the LOD.

**Table 3:** Concentrations of thyroid hormones in maternal serum and cord blood before delivery (*n* = 61).

Heading	Maternal Serum ^1^		Cord Blood	
	Median	Range	Median	Range
TSH (μIU/mL)	2.08	0.38–6.07	7.05	1.63–289.7
T_3_ (ng/dL)	140.0	82.4–277.4	56.3	35.1–84.6
T_4_ (μg/dL)	9.6	3.6–16.9	7.66	3.65–11.7
FT_4_ (ng/dL)	0.99	0.33–1.31	1.13	0.49–1.45

^1^ Reference values for thyroid hormones in Taiwan: TSH: 0.27–4.2; T_3_: 84.6–202.0; T_4_: 5.13–14.1; FT_4_: 0.93–1.7.

**Table 4:** Spearman correlation coefficients between thyroid hormones and phthalate monoesters in serum samples (*n* = 60) ^a^.

	Maternal Serum				Cord Serum			
	T_4_	T_3_	FT_4_	TSH ^c^	T_4_	T_3_	FT_4_	TSH ^c^
T_4_	-				-			
T_3_	0.53 *	-			0.15	-		
FT_4_	0.76 *	0.20	-		0.62 *	0.25	-	
TSH	0.13	0.39 *	0.16	-	0.35 *	0.03	0.21	-
MBP	−0.08	−0.11	−0.14	−0.06	**−0.23** ^+^	0.11	0.10	**−0.25** ^#^
MEHP	0.01	0.19	−0.11	0.08	−0.04	0.18	0.01	−0.07
MEP	−0.14	−0.17	−0.06	−0.13	−0.05	0.09	−0.07	0.05
Age ^b^	−0.10	−0.15	0.05	0.04	−0.09	−0.03	0.10	−0.04

^a^ *: *p* < 0.05; ^#^: *p* < 0.06; ^+^: *p* < 0.10. ^b^ Current age for pregnant women and gestational age for newborns. ^c^ TSH in cord serum and maternal serum were log-transferred.

**Table 5:** Multiple linear regression between TSH and T_4_ levels and their corresponding phthalate metabolites in cord serum (*n* = 60) ^a^.

Variables	TSH (μIU/mL)		TSH × T_4_		TSH × FT_4_		FT_4_/T_4_		T_4_ (μg/dL)	
	Estimate	*p*	Estimate	*p*	Estimate	*p*	Estimate	*p*	Estimate	*p*
Intercept	3.49	0.010	171.4	0.004	20.6	0.001	0.123	0.006	20.4	0.017
MBP_cord serum_	−0.217	0.044 *	−42.8	0.028 *	−4.49	0.075 ^#^	0.036	0.004 **	−1.71	0.036 *
Maternal age	—	—	—	—	—	—	−0.002	0.087 ^#^	0.113	0.106
Gestational age	−0.045	0.117	—	—	—	—	—	—	−0.315	−0.104
R^2^	0.087	0.044	0.092	0.028	0.054	0.075	0.171	0.005	0.115	0.046

^a^ All the parameters were log-transformed. Estimate values are beta coefficients except for R^2^; ^#^ *p* < 0.10. * *p* < 0.05. ** *p* < 0.01. —, Excluded from stepwise forward model.

## Data Availability

The data are not publicly available due to protection of subjects’ privacy and confidentiality. The data presented in this study are available on request from the first author.

## Abbreviations

BBzP	butyl benzyl phthalate
BW	birth weight
BL	birth length
DBP	di-n-butyl phthalate
DEHP	di-(2-ethylhexyl) phthalate
DEP	di-ethyl phthalate
FT4	free T4
GA	gestational age
LC-ESI/MS/MS	liquid chromatography electrospray ionization tandem mass spectrometry
LOD	limit of detection
MBP	mono-n-butyl phthalate
MBzP	monobenzyl phthalate
MDL	minimum detectable limit
MEHP	mono-2-ethylhexyl phthalate
MEP	monoethyl phthalate
MMP	monomethyl phthalate
ND	not detectable
T_3_	triiodothyronine
T_4_	thyroxine
TSH	thyroid stimulation hormone

## References

[1] Wang S.-L., Su P.-H., Jong S.-B., Guo Y.L., Chou W.-L., Päpke O. (2005). In Utero Exposure to Dioxins and Polychlorinated Biphenyls and Its Relations to Thyroid Function and Growth Hormone in Newborns. Environ. Health Perspect..

[2] Mazdai A., Dodder N.G., Abernathy M.P., Hites R.A., Bigsby R.M. (2003). Polybrominated diphenyl ethers in maternal and fetal blood samples. Environ. Health Perspect..

[3] Ghassabian A., Trasande L. (2018). Disruption in Thyroid Signaling Pathway: A Mechanism for the Effect of Endocrine-Disrupting Chemicals on Child Neurodevelopment. Front. Endocrinol..

[4] Bloom M.S., Jansing R.L., Kannan K., Rej R., Fitzgerald E.F. (2014). Thyroid hormones are associated with exposure to persistent organic pollutants in aging residents of upper Hudson River communities. Int. J. Hyg. Environ. Health.

[5] Derakhshan A., Philips E.M., Ghassabian A., Santos S., Asimakopoulos A.G., Kannan K., Kortenkamp A., Jaddoe V.W., Trasande L., Peeters R.P. (2021). Association of urinary bisphenols during pregnancy with maternal, cord blood and childhood thyroid function. Environ. Int..

[6] Haddow J.E., Palomaki G.E., Allan W.C., Williams J.R., Knight G.J., Gagnon J., O’Heir C.E., Mitchell M.L., Hermos R.J., Waisbren S.E. (1999). Maternal Thyroid Deficiency during Pregnancy and Subsequent Neuropsychological Development of the Child. N. Engl. J. Med..

[7] Pop V.J., Kuijpens J.L., Van Baar A.L., Verkerk G., Van Son M.M., De Vijlder J.J., Vulsma T., Wiersinga W.M., Drexhage H.A., Vader H.L. (1999). Low maternal free thyroxine concentrations during early pregnancy are associated with impaired psychomotor development in infancy. Clin. Endocrinol..

[8] Poppe K. (2003). Thyroid autoimmunity and hypothyroidism before and during pregnancy. Hum. Reprod. Updat..

[9] Casey B.M., Dashe J.S., Wells C.E., McIntire D.D., Leveno K.J., Cunningham F.G. (2006). Subclinical Hyperthyroidism and Pregnancy Outcomes. Obstet. Gynecol..

[10] Lischinsky J.E., Skocic J., Clairman H., Rovet J. (2016). Preliminary Findings Show Maternal Hypothyroidism May Contribute to Abnormal Cortical Morphology in Offspring. Front. Endocrinol..

[11] Samadi A., Skocic J., Rovet J.F. (2015). Children Born to Women Treated for Hypothyroidism During Pregnancy Show Abnormal Corpus Callosum Development. Thyroid.

[12] Jansen T.A., Korevaar T.I.M., Mulder T.A., White T., Muetzel R.L., Peeters R.P., Tiemeier H. (2019). Maternal thyroid function during pregnancy and child brain morphology: A time window-specific analysis of a prospective cohort. Lancet Diabetes Endocrinol..

[13] Huang P.-C., Kuo P.-L., Guo Y.-L., Liao P.-C., Lee C.-C. (2007). Associations between urinary phthalate monoesters and thyroid hormones in pregnant women. Hum. Reprod..

[14] Huang P.-C., Tsai C.-H., Liang W.-Y., Li S.-S., Huang H.-B., Kuo P.-L. (2016). Early Phthalates Exposure in Pregnant Women Is Associated with Alteration of Thyroid Hormones. PLoS ONE.

[15] Huang H.-B., Kuo P.-L., Chang J.-W., Jaakkola J.J., Liao K.-W., Huang P.-C. (2018). Longitudinal assessment of prenatal phthalate exposure on serum and cord thyroid hormones homeostasis during pregnancy—Tainan birth cohort study (TBCS). Sci. Total. Environ..

[16] Kuo F.-C., Su S.-W., Wu C.-F., Huang M.-C., Shiea J., Chen B.-H., Chen Y.-L., Wu M.-T. (2015). Relationship of Urinary Phthalate Metabolites with Serum Thyroid Hormones in Pregnant Women and Their Newborns: A Prospective Birth Cohort in Taiwan. PLoS ONE.

[17] Romano M.E., Eliot M.N., Zoeller R.T., Hoofnagle A.N., Calafat A.M., Karagas M.R., Yolton K., Chen A., Lanphear B.P., Braun J.M. (2018). Maternal urinary phthalate metabolites during pregnancy and thyroid hormone concentrations in maternal and cord sera: The HOME Study. Int. J. Hyg. Environ. Health.

[18] Wang Y., Zhu H., Kannan K. (2019). A Review of Biomonitoring of Phthalate Exposures. Toxics.

[19] Huang P.-C., Waits A., Chen H.-C., Chang W.-T., Jaakkola J.J., Huang H.-B. (2020). Mediating role of oxidative/nitrosative stress biomarkers in the associations between phthalate exposure and thyroid function in Taiwanese adults. Environ. Int..

[20] Yuan S., Liu C., Liao C., Chang B. (2002). Occurrence and microbial degradation of phthalate esters in Taiwan river sediments. Chemosphere.

[21] Bolt H.M., Koch H.M. (2004). Di(2-ethylhexyl)phthalate (DEHP) metabolites in human urine and serum after a single oral dose of deuterium-labelled DEHP. Arch. Toxicol..

[22] Loftus C.T., Bush N.R., Day D.B., Ni Y., Tylavsky F.A., Karr C.J., Kannan K., Barrett E.S., Szpiro A.A., Sathyanarayana S. (2021). Exposure to prenatal phthalate mixtures and neurodevelopment in the Conditions Affecting Neurocognitive Development and Learning in Early childhood (CANDLE) study. Environ. Int..

[23] Santos S., Sol C.M., van Janssens C.Z., Philips E.M., Asimakopoulos A.G., Martinez-Moral M.-P., Kannan K., Jaddoe V.W., Trasande L. (2021). Maternal phthalate urine concentrations, fetal growth and adverse birth outcomes. A population-based prospective cohort study. Environ. Int..

[24] Li A.J., Martinez-Moral M.-P., Al-Malki A.L., Al-Ghamdi M.A., Al-Bazi M.M., Kumosani T.A., Kannan K. (2019). Mediation analysis for the relationship between urinary phthalate metabolites and type 2 diabetes via oxidative stress in a population in Jeddah, Saudi Arabia. Environ. Int..

[25] Adibi J.J., Perera F.P., Jedrychowski W., Camann D.E., Barr D., Jacek R., Whyatt R.M. (2003). Prenatal exposures to phthalates among women in New York City and Krakow, Poland. Environ. Health Perspect..

[26] Arbuckle T.E., Davis K., Marro L., Fisher M., Legrand M., Leblanc A., Gaudreau E., Foster W.G., Choeurng V., Fraser W.D. (2014). Phthalate and bisphenol A exposure among pregnant women in Canada—Results from the MIREC study. Environ. Int..

[27] Guth M., Pollock T., Fisher M., Arbuckle T.E., Bouchard M.F. (2021). Concentrations of urinary parabens and reproductive hormones in girls 6–17 years living in Canada. Int. J. Hyg. Environ. Health.

[28] Lin S., Ku H.-Y., Su P.-H., Chen J.-W., Huang P.-C., Angerer J., Wang S.-L. (2011). Phthalate exposure in pregnant women and their children in central Taiwan. Chemosphere.

[29] Hauser R., Meeker J.D., Park S., Silva M.J., Calafat A.M. (2004). Temporal Variability of Urinary Phthalate Metabolite Levels in Men of Reproductive Age. Environ. Health Perspect..

[30] Latini G., De Felice C., Presta G., Del Vecchio A., Paris I., Ruggieri F., Mazzeo P. (2003). In utero exposure to di-(2-ethylhexyl)phthalate and duration of human pregnancy. Environ. Health Perspect..

[31] Jensen M.S., Nørgaard-Pedersen B., Toft G., Hougaard D.M., Bonde J.P., Cohen A., Thulstrup A.M., Ivell R., Anand-Ivell R., Lindh C.H. (2012). Phthalates and Perfluorooctanesulfonic Acid in Human Amniotic Fluid: Temporal Trends and Timing of Amniocentesis in Pregnancy. Environ. Health Perspect..

[32] Calafat A.M., Brock J.W., Silva M.J., Gray L.E., Reidy J.A., Barr D.B., Needham L.L. (2006). Urinary and amniotic fluid levels of phthalate monoesters in rats after the oral administration of di(2-ethylhexyl) phthalate and di-n-butyl phthalate. Toxicology.

[33] Guo J., Wu M., Gao X., Chen J., Li S., Chen B., Dong R. (2020). Meconium Exposure to Phthalates, Sex and Thyroid Hormones, Birth Size and Pregnancy Outcomes in 251 Mother–Infant Pairs from Shanghai. Int. J. Environ. Res. Public Health.

[34] Hinton R.H., Mitchell F.E., Mann A., Chescoe D., Price S.C., Nunn A., Grasso P., Bridges J.W. (1986). Effects of phthalic acid esters on the liver and thyroid. Environ. Health Perspect..

[35] Price S.C., Chescoe D., Grasso P., Wright M., Hinton R.H. (1988). Alterations in the thyroids of rats treated for long periods with di-(2-ethylhexyl) phthalate or with hypolipidaemic agents. Toxicol. Lett..

[36] Poon R., Lecavalier P., Mueller R., Valli V., Procter B., Chu I. (1997). Subchronic oral toxicity of di-n-octyl phthalate and di(2-ethylhexyl) phthalate in the rat. Food Chem. Toxicol..

[37] Sugiyama S.-I., Shimada N., Miyoshi H., Yamauchi K. (2005). Detection of Thyroid System–Disrupting Chemicals Using in Vitro and in Vivo Screening Assays in Xenopus laevis. Toxicol. Sci..

[38] Pereira C., Mapuskar K., Rao C.V. (2007). A two-generation chronic mixture toxicity study of Clophen A60 and diethyl phthalate on histology of adrenal cortex and thyroid of rats. Acta Histochem..

[39] Shen O., Wu W., Du G., Liu R., Yu L., Sun H., Han X., Jiang Y., Shi W., Hu W. (2011). Thyroid Disruption by Di-n-Butyl Phthalate (DBP) and Mono-n-Butyl Phthalate (MBP) in Xenopus laevis. PLoS ONE.

[40] Liu C., Zhao L., Wei L., Li L. (2015). DEHP reduces thyroid hormones via interacting with hormone synthesis-related proteins, deiodinases, transthyretin, receptors, and hepatic enzymes in rats. Environ. Sci. Pollut. Res..

[41] Ye H., Ha M., Yang M., Yue P., Xie Z., Liu C. (2017). Di2-ethylhexyl phthalate disrupts thyroid hormone homeostasis through activating the Ras/Akt/TRHr pathway and inducing hepatic enzymes. Sci. Rep..

[42] Waits A., Chen H.-C., Kuo P.-L., Wang C.-W., Huang H.-B., Chang W.-H., Shih S.-F., Huang P.-C. (2020). Urinary phthalate metabolites are associated with biomarkers of DNA damage and lipid peroxidation in pregnant women—Tainan Birth Cohort Study (TBCS). Environ. Res..

[43] Breous E., Wenzel A., Loos U. (2005). The promoter of the human sodium/iodide symporter responds to certain phthalate plasticisers. Mol. Cell. Endocrinol..

[44] Takser L., Mergler D., Baldwin M., De Grosbois S., Smargiassi A., Lafond J. (2005). Thyroid Hormones in Pregnancy in Relation to Environmental Exposure to Organochlorine Compounds and Mercury. Environ. Health Perspect..

[45] Johns L.E., Ferguson K.K., McElrath T.F., Mukherjee B., Meeker J.D. (2016). Associations between Repeated Measures of Maternal Urinary Phthalate Metabolites and Thyroid Hormone Parameters during Pregnancy. Environ. Health Perspect..

[46] Minatoya M., Jima S.N., Sasaki S., Araki A., Miyashita C., Ikeno T., Nakajima T., Goto Y., Kishi R. (2016). Effects of prenatal phthalate exposure on thyroid hormone levels, mental and psychomotor development of infants: The Hokkaido Study on Environment and Children’s Health. Sci. Total. Environ..

[47] Yao H.-Y., Han Y., Gao H., Huang K., Ge X., Xu Y.-Y., Xu Y.-Q., Jin Z.-X., Sheng J., Yan S.-Q. (2016). Maternal phthalate exposure during the first trimester and serum thyroid hormones in pregnant women and their newborns. Chemosphere.

[48] Silva M.J., Samandar E., Preau J.L., Reidy J.A., Needham L.L., Calafat A.M. (2005). Automated Solid-Phase Extraction and Quantitative Analysis of 14 Phthalate Metabolites in Human Serum using Isotope Dilution-High-Performance Liquid Chromatography-Tandem Mass Spectrometry. J. Anal. Toxicol..

[49] Salazar-Martinez E., Romano-Riquer P., Yanez-Marquez E., Longnecker M.P., Hernandez-Avila M. (2004). Anogenital distance in human male and female newborns: A descriptive, cross-sectional study. Environ. Health.

[50] Aggarwal N., Razvi S. (2013). Thyroid and Aging or the Aging Thyroid? An Evidence-Based Analysis of the Literature. J. Thyroid. Res..

[51] Meeker J.D., Calafat A.M., Hauser R. (2007). Di(2-ethylhexyl) Phthalate Metabolites May Alter Thyroid Hormone Levels in Men. Environ. Health Perspect..

[52] Eltom A., Eltom M., Idris M., Gebre-Medhin M. (2001). Thyroid function in the newborn in relation to maternal thyroid status during labour in a mild iodine deficiency endemic area in Sudan. Clin. Endocrinol..

[53] James D.K., David K. (2006). High Risk Pregnancy Management Options.

[54] Ozdemir H., Akman I., Coskun S., Demirel U., Turan S., Bereket A., Bilgen H., Ozek E. (2013). Maternal Thyroid Dysfunction and Neonatal Thyroid Problems. Int. J. Endocrinol..

[55] Albro P.W., Corbett J.T., Schroeder J.L., Jordan S., Matthews H.B. (1982). Pharmacokinetics, interactions with macromolecules and species differences in metabolism of DEHP. Environ. Health Perspect..

[56] Peck C.C., Albro P.W. (1982). Toxic potential of the plasticizer Di(2-ethylhexyl) phthalate in the context of its disposition and metabolism in primates and man. Environ. Health Perspect..

[57] Egestad B., Green G., Sjöberg P., Klasson-Wehler E., Gustafsson J. (1996). Chromatographic fractionation and analysis by mass spectrometry of conjugated metabolites of bis(2-ethylhexyl)phthalate in urine. J. Chromatogr. B Biomed. Sci. Appl..

[58] Frederiksen H., Kuiri-Hänninen T., Main K.M., Dunkel L., Sankilampi U. (2014). A Longitudinal Study of Urinary Phthalate Excretion in 58 Full-Term and 67 Preterm Infants from Birth through 14 Months. Environ. Health Perspect..

[59] Main K.M., Mortensen G.K., Kaleva M.M., Boisen K.A., Damgaard I.N., Chellakooty M., Schmidt I.M., Suomi A.-M., Virtanen H.E., Petersen D.V.H. (2006). Human Breast Milk Contamination with Phthalates and Alterations of Endogenous Reproductive Hormones in Infants Three Months of Age. Environ. Health Perspect..

[60] Pan G., Hanaoka T., Yoshimura M., Zhang S., Wang P., Tsukino H., Inoue K., Nakazawa H., Tsugane S., Takahashi K. (2006). Decreased Serum Free Testosterone in Workers Exposed to High Levels of Di-n-butyl Phthalate (DBP) and Di-2-ethylhexyl Phthalate (DEHP): A Cross-Sectional Study in China. Environ. Health Perspect..

[61] Meeker J.D., Ferguson K.K. (2014). Urinary phthalate metabolites are associated with decreased serum testosterone in men, women, and children from NHANES 2011–2012. J. Clin. Endocrinol. Metab..

[62] ATSDR (1995). Toxicological Profile for Diethyl Phthalate.

[63] Radke E.G., Galizia A., Thayer K.A., Cooper G.S. (2019). Phthalate exposure and metabolic effects: A systematic review of the human epidemiological evidence. Environ. Int..

[64] Warner G.R., Li Z., Houde M.L., Atkinson C.E., Meling D.D., Chiang C., Flaws J.A. (2019). Ovarian Metabolism of an Environmentally Relevant Phthalate Mixture. Toxicol. Sci..

[65] Janjua N.R., Mortensen G.K., Andersson A.-M., Kongshoj B., Wulf H.C. (2007). Systemic Uptake of Diethyl Phthalate, Dibutyl Phthalate, and Butyl Paraben Following Whole-Body Topical Application and Reproductive and Thyroid Hormone Levels in Humans. Environ. Sci. Technol..

